# Sounds from the Consulting Room

**Published:** 1883-09

**Authors:** 


INJURIOUS ALGAE.--In a paper on some Algæ of Minnesota supposed to be poisonous, Prof. J. C. Arthur gives an account of a species of Rivularia infesting the water of ponds at Waterville, Minnesota, and supposed to be the cause of death or injury to cattle. He also describes the condition of Lake Phalen, near St. Paul, in which he found several species of Nostochaceæ.—(*Science.*)

